# Lateralization of delta band power in magnetoencephalography (MEG) in patients with unilateral focal epilepsy and its relation to verbal fluency

**DOI:** 10.1002/brb3.3257

**Published:** 2023-09-26

**Authors:** Annika Melissa Süß, Marion Hug, Nadine Conradi, Ricardo Kienitz, Felix Rosenow, Stefan Rampp, Nina Merkel

**Affiliations:** ^1^ Epilepsy Center Frankfurt Rhine‐Main Center of Neurology and Neurosurgery University Hospital Frankfurt Frankfurt am Main Germany; ^2^ Department of Neurology University Hospital Frankfurt and Goethe University Frankfurt am Main Germany; ^3^ Department of Neurosurgery University Hospital Erlangen Erlangen Germany; ^4^ Department of Neurosurgery University Hospital Halle (Saale) Halle (Saale) Germany; ^5^ Ernst Strüngmann Institute for Neuroscience in Cooperation with Max Planck Society Frankfurt am Main Germany; ^6^ LOEWE Center for Personalized Translational Epilepsy Research (CePTER), Goethe University Frankfurt Frankfurt am Main Germany

**Keywords:** delta band power, focal epilepsy, MEG, verbal fluency

## Abstract

**Introduction:**

Delta power is a clinically established biomarker for abnormal brain processes. However, in patients with unilateral focal epilepsy (FE) it is still not well understood, how it relates to the epileptogenic zone and to neurocognitive functioning. The aim of the present study was thus to assess how delta power relates to the affected hemisphere, whether lateralization strength differs between the patients, and how changes in delta power correlate with cognitive functioning.

**Method:**

We retrospectively studied patients with left (LFE) and right FE (RFE) who had undergone a resting‐state magnetoencephalography measurement. We computed global and hemispheric delta power and lateralization indices and examined whether delta power correlates with semantic and letter verbal fluency (former being a marker for language and verbal memory, latter for executive functions) in 26 FE patients (15 LFE, 11 RFE) and 10 healthy controls.

**Results:**

Delta power was increased in FE patients compared to healthy controls. However, the increase across hemispheres was related to the site of the epileptic focus: On group level, LFE patients showed higher delta power in both hemispheres, whereas RFE patients primarily exhibited higher delta power in the ipsilateral right hemisphere. Both groups showed co‐fluctuations of delta power between the hemispheres. Besides, delta power correlated negatively only with letter verbal fluency.

**Conclusion:**

The findings confirm and provide further evidence that delta power is a marker of pathological activity and abnormal brain processes in FE. Delta power dynamics differ between patient groups, indicating that delta power could offer additional diagnostic value. The negative association of delta power and letter verbal fluency suggests that executive dysfunctions are related to low frequency abnormalities.

## Graphical abstract

We studied patients with left and right focal epilepsy, and assessed how resting state magnetoencephalogaphy delta power lateralization relates to the affected hemisphere, how it differs between patients, and how changes in delta power correlate with cognitive functioning. Our findings provide further evidence that delta power is a marker of pathological activity and abnormal brain processes in focal epilepsy. Differences found in delta power dynamics between patient groups indicate that delta power could offer additional diagnostic value in a patient group specific manner. A negative association between delta power and letter verbal fluency suggests that executive dysfunctions are related to low frequency abnormalities.

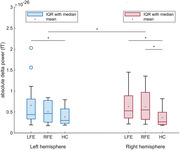

## 1 INTRODUCTION

With a relative frequency of 60%, focal epilepsies (FE) are the most common form of epilepsy (Rosenow & Lüders, 2001). Despite their focal nature, recent evidences point to their role in the context of a more (global) network disorder (Englot et al., 2016). This is because epileptic activity is known to interfere with spontaneous brain activity in resting‐state networks, also referred to as intrinsic functional connectivity networks (Cataldi et al., 2013; Haneef et al., 2012). The language network (Trimmel et al., 2018; Waites et al., 2006) and the attention‐ and executive control network are two examples of such connectivity networks (Cataldi et al., 2013). Thus, neurophysiological markers of epileptic hyperexcitability and related cognitive dysfunction should be investigated not only locally, but also globally.

Focal and global neurophysiological markers of epilepsy can be measured with electroencephalography (EEG) and magnetoencephalography (MEG) and appear as interictal epileptiform discharges or as slowing of background activity. Interictal epileptiform discharges are epilepsy specific phenomena that occur regionally as spikes or sharp waves and indicate the irritative zone, which is in turn spatially related to the epileptogenic zone in a FE (Rosenow & Lüders, 2001). Slowing of the background activity, such as increases in the delta band (1–4 Hz), on the other hand, are not epilepsy specific per se, but rather an unspecific indicator of neuronal pathology (Lin et al., 2018) that can be present in patients with FE and might indicate the lateralization and localization of the epileptogenic zone (Di Gennaro et al., 2003; Englot et al., 2016; Ishibashi et al., 2002; Laufs et al., 2006; Lin et al., 2018; Pellegrino et al., 2017). However, its exact underlying pathophysiology and the extent of its contribution to the diagnosis is still unclear (Knyazev, 2012).

Physiologically, delta band oscillations are high amplitude low frequency oscillations that are assumed to be an evolutionary ancient phenomenon (Knyazev, 2012). They occur during early brain developmental stages and their incidence slowly decreases in favor of higher frequencies during brain development (Clarke et al., 2001; Kulandaivel & Holmes, 2011)—in parallel with the development of more complex cognitive processes, such as language and higher cognitive functions (Paz‐Alonso et al., 2014). As the decrease of delta power during development cooccurs with a decrease of synaptic density, it has been suggested that it plays a role in cognitive pruning and efficient information processing (Feinberg & Campbell, 2010). Delta oscillations are furthermore known as physiological signs of deep sleep (Knyazev, 2012).

Increased delta power in developed brains in awake state, however, often indicates an underlying pathology and has been associated with decreased cognitive functioning (Fernández et al., 2002; Ostrowski et al., 2021; Rommel et al., 2017). Delta band increases often co‐occur with structural abnormalities, for example, in brain tumors (Kamada et al., 2001) and with changes in gray and white matter (Brigo, 2011), but can also be found without any structural abnormalities (Knyazev, 2012). Decreased delta power has been found in autism, whereas increased delta power has been shown to occur in depression, schizophrenia, obsessive compulsive disorder, anxiety disorder, and Alzheimer dementia (Fernández et al., 2002; Newson & Thiagarajan, 2018).

In epilepsy, increases in delta power are known to occur in many patients with temporal (TLE) and extratemporal lobe epilepsy, as regional or global, intermittent, or continuous slowing of background activity (Fisher et al., 2017; Kamada et al., 2001). Although several studies indicate a correlation with lateralization and localization of the epileptogenic zone (Di Gennaro et al., 2003; Englot et al., 2016; Ishibashi et al., 2002; Lin et al., 2018; Pellegrino et al., 2017; Tao et al., 2011), other recent findings indicate that delta band activity also occurs bilaterally and distantly to the epileptogenic zone (Lundstrom et al., 2019; Sachdev et al., 2015). Thus, the exact diagnostic relevance is still uncertain. Specifically, it remains unclear, whether delta band alterations encompass the entire brain, how patients’ delta band dynamics differ between hemispheres and whether they are specific for selective patient groups. Furthermore, it is unclear, whether and how delta band power is related to cognitive functioning in FE patients.

The aim of the present study was therefore to investigate delta band power in resting‐state MEG in patients with unilateral left (LFE) or right focal epilepsy (RFE) in comparison with healthy controls. We hypothesized that delta power in unilateral FE patients is increased compared to healthy controls. This might be present locally and close to the epileptic focus or a rather global phenomenon and extend to the contralateral hemisphere. Furthermore, as delta band activity has been associated with several cognitive processes (Fernández et al., 2002; Ostrowski et al., 2021; Rommel et al., 2017), we investigated, whether delta power is correlated with verbal fluency—a measure of cognitive functioning that comprises aspects of language and verbal memory in the case of semantic verbal fluency or more executive functions in the case of letter verbal fluency (Amunts et al., 2020; Metternich et al., 2014).

## 2 SUBJECTS AND METHODS

### 2.1 Subjects

Forty patients with pharmacoresistant FE who had undergone presurgical evaluation in our epilepsy center and underwent both MEG and neuropsychological assessment were initially considered for the study. Neuropsychological assessment was a mandatory procedure during the presurgical epilepsy evaluation, whereas patients underwent a MEG recording when additional information about the focus localization was required, often in MRI‐negative cases.

Fourteen patients were excluded from the analyses due to (1) seizures during recordings (*n* = 1), (2) high levels of movement artefacts (*n* = 3), (3) uncertain epilepsy diagnosis (*n* = 5), (4) mental disability (*n* = 1), (5) age below 16 years (*n* = 2), and (6) bilateral lesions in MRI (*n* = 2). Finally, 26 patients (11 women, 15 men) with a mean age of 34.9 years (*SD* = 12.3, *median* = 31.5, *range*: 17–58 years) were included and studied retrospectively. The final epilepsy diagnosis at the beginning of the data analyses in 10/2020 was used to classify the patients either as having LFE or RFE. The epilepsy syndrome was classified by neurologists during the patient's stay in the video‐EEG monitoring unit by means of seizure type as well as the EEG and MRI (and sometimes PET) findings and the classification was based on the latest recommendations of the International League Against Epilepsy (ILAE; Fisher et al., 2017) and the integrated epilepsy classification (Rosenow et al., 2020). Fifteen of the patients (57.7%) had LFE and 11 patients (42.3%) had RFE. Furthermore, as 20 patients had valid language lateralization results (based on either fTCD [functional transcranial Doppler ultrasound] or Wada test) and verbal fluency test results, the neuropsychological data of 20 out of 26 patients included in the previous analyses were evaluated retrospectively. Demographic features, as well as clinical and neuropsychological characteristics, can be found in Tables 1 and 2. Additionally, 10 healthy controls (4 women, 6 men) with a mean age of 26.5 years (*SD* = 3.3, *median* = 26.5, *range*: 21–38 years) were used for comparison. All subjects gave their written informed consent for the use of their anonymized data for scientific purposes and publication.

**TABLE 1:** Demographic, clinical, and neuropsychological characteristics of the 26 patients with unilateral focal epilepsy (FE).

	Patients with LFE (*n* = 15)	Patients with RFE (*n* = 11)
**Gender**		
− Female	6 (40.0%)	5 (45.5%)
− Male	9 (60.0%)	6 (54.5%)
**Age**	33.1 (*SD* = 11.7, *median* = 27.0, range: 17–53)	37.4 (*SD* = 13.3, *median* = 37.0, range: 19–58)
**Age at initial diagnosis (onset)**	22.3 (*SD* = 10.8, *median* = 20.0, range: 10–44)	23.7 (*SD* = 10.8, *median* = 21.0, range: 1–38)
**Duration (years)**	10.8 (*SD* = 11.5, *median* = 7.0, range: 1–41)	13.6 (*SD* = 16.7, *median* = 7.0, range: 1–51)
**Localization of epileptogenic zone**		
Language relevant regions	12 (80.0%)	9 (81.8%)
− Temporal	8 (53.3%)	1 (9.1%)
− Frontal	3 (20.0%)	7 (63.6%)
− Frontotemporal	1 (6.7%)	1 (9.1%)
Language nonrelevant regions	3 (20.0%)	2 (18.2%)
**Structural lesion in the epileptic hemisphere**		
− Yes	11 (73.3%)	3 (27.3%)
− No	4 (26.7%)	8 (72.7%)
**Language lateralization**		
− Left	11 (73.3%)	8 (72.7%)
− Right	1 (6.7%)	1 (9.1%)
− Unclear or not tested	3 (20.0%)	2 (18.2%)
**Handedness**		
− Left	0 (0.0%)	0 (0.0%)
− Right	11 (73.3%)	11 (100.0%)
− Unknown	4 (26.7%)	0 (0.0%)
**Verbal fluency** **(** ***PR*** **)**		
− Lexical	43.9 (*SD* = 28.8, *median* = 50.0, range: 10–84)	41.1 (*SD* = 32.0, *median* = 25.1, range: 10–90)
− Semantic	45.6 (*SD* = 23.4, *median* = 50.0, range: 10–75)	43.5 (*SD* = 22.1, *median* = 50.1, range: 16–75)

*Note*: Handedness was determined by means of the *Edinburgh Handedness Inventory* (EHI); language lateralization was determined by means of functional transcranial Doppler sonography (fTCD) or Wada test; *n*, number.

Abbreviations: FE, focal epilepsy; *PR*, percentile rank; *SD*, standard deviation.

**TABLE 2:** Epilepsy‐specific data of the individual patients with unilateral left (LFE) or right focal epilepsy (RFE).

Pat. no.	Localization of the epileptogenic zone	Etiology	ASM
	**Left hemisphere**		
1	Temporal DD insular	FCD frontal	ZNS, LTG
2	Fronto‐central	FCD frontal	LTG
3	Temporal	Unknown (no lesion)	LEV
4	Temporal	HS	BRV, LCM, med. cannabis
5	Temporal	PVNH and HS	LTG, LEV
6	Frontal (DD mesial)	Unknown (no lesion)	LEV, LCM
7	Frontal	FCD frontal	OXC, ZNS
8	Temporal	Amygdala hyperplasia	LEV
9	Mesio‐frontal	FCD frontal	BRV, LCM, LEV, LTG
10	Temporal	Unknown (no lesion)	LTG
11	Temporal	Unknown (no lesion)	LTG, LEV
12	Temporal	Amygdala dysplasia	LTG, LCM
13	Frontotemporal	Heterotopia frontal	BRV, VPA, PGB
14	Central	Unknown	LTG
15	Mesial parieto‐occipital	FCD frontal	CBZ, BRV, PER
	**Right hemisphere**		
16	Temporal	Unknown (no lesion)	LTG, LEV
17	Frontal	Unknown (no lesion)	LEV, LCM
18	Frontal or temporo‐occipital	Unknown (no lesion)	VPA, BRV, CLB, Mirtazapin,
19	Frontal	Unknown (no lesion)	LCM, BRV
20	Frontopolar	FCD frontal	LTG
21	Central	Cavernoma central	BRV, ESL
22	Frontal	Unknown (no lesion)	BRV, LTG, PER
23	Frontal	FCD frontal	LEV, LCM
24	Frontal	FCD frontal	PB, CLB, LEV, LTG
25	Frontotemporal	Unknown (no lesion)	LTG, LEV
26	Frontal	Polymicrogyria frontal	LTG, CBZ

Abbreviations: ASM, anti‐seizure medication; BRV, brivaracetam; CBZ, carbamazepine; CLB, clobazam; DD, differential diagnosis; ESL, eslicarbazepinacetate; FCD, focal cortical dysplasia; HS, hippocampal sclerosis; LCM, lacosamide; LEV, levetriracetam; LTG, lamotrigine; med, medical; OXC, oxcarbazepin; Pat. no., patient number; PB, phenobarbital; PER, perampanel; PGB, pregabalin; PVNH, periventricular nodular heterotopia; VPA, sodiumvalproate; ZNS, zonisamide.

### 2.2 MEG recording

MEG was recorded using standard procedures as recommended in Gross et al. (2013) and Hari et al. (2018). The MEG recordings were conducted in the Brain Imaging Center of the Goethe University Frankfurt am Main between 07/2017 and 11/2019. All subjects underwent a briefing outside the MEG chamber, received fully metal free surgery clothing, and were provided with electrodes for electrooculography (EOG) for recording horizontal and vertical eye movements and electrodes for electrocardiography (ECG) for recording heartbeat as well as ground electrodes. The electrodes were placed at previously cleaned spots using ultrasound gel. EOG electrodes were positioned distal to the eyelid of the left and right eye and in upper and lower line with the pupil. ECG electrodes were placed at the left and right clavicular. The electrode impedances were tested using an electrode impedance meter and were kept under 10 kΩ. Typical warning signs of seizures, special requirements in terms of seizure prevention and handling of potential seizures as well as patient's individual emergency medication were additionally evaluated.

MEG resting state was recorded in a sitting position with eyes closed. The subject's head was brought as close as possible to the dewar and was stabilized using small, soft cushions. Subjects were instructed to remain still and relaxed, to keep eyes closed, and to avoid eye and head movements. Head position was continuously monitored via three localization coils (nasion as well as left and right in‐ear position). For patient's safety, the inside of the chamber was monitored using an eye‐tracking camera. It was further communicated via an intercom system between the runs to maintain vigilance. For the patients’ safety a medical doctor was close‐by and on call while data was recorded. All data was collected with a whole head CTF 275‐channel MEG‐system (Omega 2005, VSM MedTech) in a magnetically shielded chamber. The MEG signal was sampled with 1200 Hz using synthetic gradiometers of third order, high pass (0.1 Hz), and low pass (300 Hz) filters as well as anti‐aliasing fourth order Butterworth filter. Data of 10 min length was recorded.

### 2.3 Neuropsychological assessment of verbal fluency and language lateralization

The neuropsychological assessment was performed by trained neuropsychologists as part of the clinical epilepsy diagnostic routine and the results were retrospectively used for the study. The neuropsychological assessment followed the recommendations by the Neuropsychological Commission of the German ILAE Chapter and the Austrian, German and Swiss Working Group on Presurgical Epilepsy Diagnosis and Epilepsy Surgery (Brückner, 2012) and aimed at evaluating cognitive functions, such as language, memory, attention, and executive functions (Conradi et al., 2020). During the neuropsychological assessment, verbal fluency was evaluated in two different tasks of the Regensburger Wortflüssigkeitstest (RWT; Aschenbrenner et al., 2000): a letter and a semantic verbal fluency task. As part of the verbal fluency tasks, patients were asked to generate as many words as possible within one minute using (1) the initial letter “S” (letter verbal fluency) and (2) the category “animals” (semantic verbal fluency). Language lateralization was determined by means of functional transcranial Doppler sonography (fTCD) using a word generation paradigm (letter verbal fluency) or alternatively by means of the Wada test as described previously (Knake et al., 2003).

### 2.4 Preprocessing of MEG data

MEG data was processed using MATLAB R2019b (The MathWorks Inc.) and the open‐source MATLAB toolbox FieldTrip (Version 20191111; Oostenveld et al., 2011) and was based on recommendation of Gross et al. (2013) and the FieldTrip Community (http://fieldtriptoolbox.org). First, data was cleaned from artefacts. All datasets were visually inspected to check for plausibility and data quality. System‐related SQUID sensor jump artefacts were removed by excluding the respective sensors (maximally three per subject). To minimize head‐movement related errors, the mean head position was determined for each subject and each trial. Only trials in which the head position did not deviate >10 mm (patients) or >5 mm (controls) from the mean head position were considered for further analysis. The raw data was high‐pass filtered at 1 Hz and low‐pass filtered at 90 Hz. Next, data was down sampled to 300 Hz and independent component analysis was performed using the extended InfoMax Algorithmus (Runica) implemented in FliedTrip to identify eye blink, eye movement, and heartbeat artefacts based on the spatial distribution of the signal (per time bin) as well as the morphology. Afterward, the cleaned data was segmented into nonoverlapping 5‐s‐long trials. Last, trials that still had higher than plausible variance (>3 *SD*) were identified and excluded, which resulted in 114–120 trials per person, corresponding to approximately 10 min of data.

### 2.5 Time–frequency analysis

The preprocessed trial data was transformed into the time–frequency space, using fast Fourier transformation as implemented in FieldTrip. Using van‐Hann‐windows of 300 ms length and 50 ms sample frequency, the power was computed between 1 and 30 Hz for each trial and sensor in each subject separately. For later analysis, averaging was performed over all sensors of one hemisphere (left and right) for each person in all groups (healthy controls and FE patients; LFE and RFE), yielding subject averages. The power within the delta band, that is, the absolute power between 1 and 4 Hz, was extracted for each trial and was then averaged over frequency and over all channels (global delta power) or over frequency and over the channels of one hemisphere (lateralized delta power) for each patient and each group separately. Absolute delta power was therefore defined as the amount of MEG activity in the delta band independent of the activity in other frequency bands. Data was then further processed in MATLAB R2019b/R2020b (The MathWorks Inc.) and exported to SPSS Statistics 26 (IBM Corporation).

### 2.6 Statistical analyses

Statistical analyses were performed using SPSS Statistics 26 (IBM Corporation). Due to the small and unequal sample sizes across groups, nonparametric tests were used.

To control for confounding variables, (1, 2) Chi‐square, (3) Spearman rank, and (4) Mann–Whitney *U* tests were performed to test for influence due to (1) sex, (2) epilepsy type and duration, (3) age, and (4) psychiatric comorbidity (depression, anxiety disorder, substance abuse [alcohol or cannabis], ADHD, and/or additional psychogenic non‐epileptic seizures; *n*_LFE_ = 5, *n*_RFE_ = 3). Group differences regarding demographic and clinical data were tested using Kruskal–Wallis or Mann–Whitney *U* tests.

The lateralizing value of delta power was evaluated by examining inter‐ and intraindividual hemispheric differences in delta power between FE patients and healthy controls and by computing delta power lateralization indices (LI). Global and hemispheric delta power differences between FE patients (*n* = 26) and healthy controls (*n* = 10), between patients with LFE (*n* = 15) and healthy controls, between patients with RFE (*n* = 11) and healthy controls, as well as between patients with LFE and RFE were tested using Mann–Whitney *U* tests. Differences in delta power between the hemispheres in patients with LFE as well as RFE were tested using the Wilcoxon signed rank test. To additionally evaluate the lateralization value of delta power in each patient, LIs were calculated using an established formula (compare Lin et al., 2018):

(1) $$\mathrm{LI}\;=\;\frac{{\mathrm{DP}}_{\mathrm{LH}}-{\mathrm{DP}}_{\mathrm{RH}}}{{\mathrm{DP}}_{\mathrm{LH}}+{\mathrm{DP}}_{\mathrm{RH}}}$$

where DP represents the average absolute delta power in either the left (LH) or the right hemisphere (RH). The resulting LI can range from −1 to 1, indicating either right (LI < −.05) or left (LI >.05) lateralization, or no lateralization/bilateral occurrence (LI from −.05 to .05; compare Lin et al., 2018). For explorative purposes, the co‐fluctuation of delta power between hemisphere ipsi‐ and contralateral to the epileptogenic zone was additionally examined using Spearman rank correlations.

Furthermore, after regrouping the delta power values considering language dominance (delta power in the language dominant and nondominant hemisphere), the relation between delta power and verbal fluency was assessed in the total group of FE patients (LFE and RFE combined) using the language lateralization results and the education‐ and age‐corrected values (percentile ranks) and by calculating Spearman rank correlations.

All statistical tests were performed as one‐ or two‐sided tests and the significance level for all analyses was set to *p* < .05. To avoid statistical type two errors and consequently overlook small, but potential clinically significant effects in small sample sizes and thus practical relevance (Nakagawa, 2004), adjustment of error probability (i.e., Bonferroni correction) was foregone and instead, effect sizes were computed as recommended in Fritz et al. (2012). The effect sizes were computed using following equation:

(2) $$r\;=\;\left|\frac{z}{\sqrt{n}}\right|$$

As recommended in Fritz et al. (2012), effect sizes [correlations] were rated as large [strong] in cases of *r* ≥ .50, moderate in cases of *r* ≥ .30 and small [weak] in cases of *r* ≥ .10.

## 3 RESULTS

### 3.1 Demographic and clinical characteristics

There were no significant differences between patient groups (LFE and RFE) in terms of gender (χ^2^(2) = 0.094, *p* > .05), epilepsy type (χ^2^(6) = 9.658, *p* > .05), and age (χ^2^(2) = 3.944, *p* > .05; compare Tables 1 and 2). Furthermore, there was no correlation between age and duration of epilepsy and delta power when checking for all subjects combined (age: *r_s_* = –.14, *p* > .05; duration: *r_s_* = –.15, *p* > .05). Between patient groups, there were no differences in age of initial diagnosis (onset; *U* = 88, *Z* = −0.372, *p* > .05) and duration of the disease (*U* = 86, *Z* = −0.466, *p* > .05). Delta power did not differ significantly between patients with and without psychiatric disorders in neither the left (*U* = 60, *Z* = −0.376, *p* > .05) nor the right hemisphere (*U* = 56, Z = −0.607, *p* > .05).

### 3.2 Global and hemispheric delta power

#### 3.2.1 Patients versus healthy controls

**FIGURE 1:**
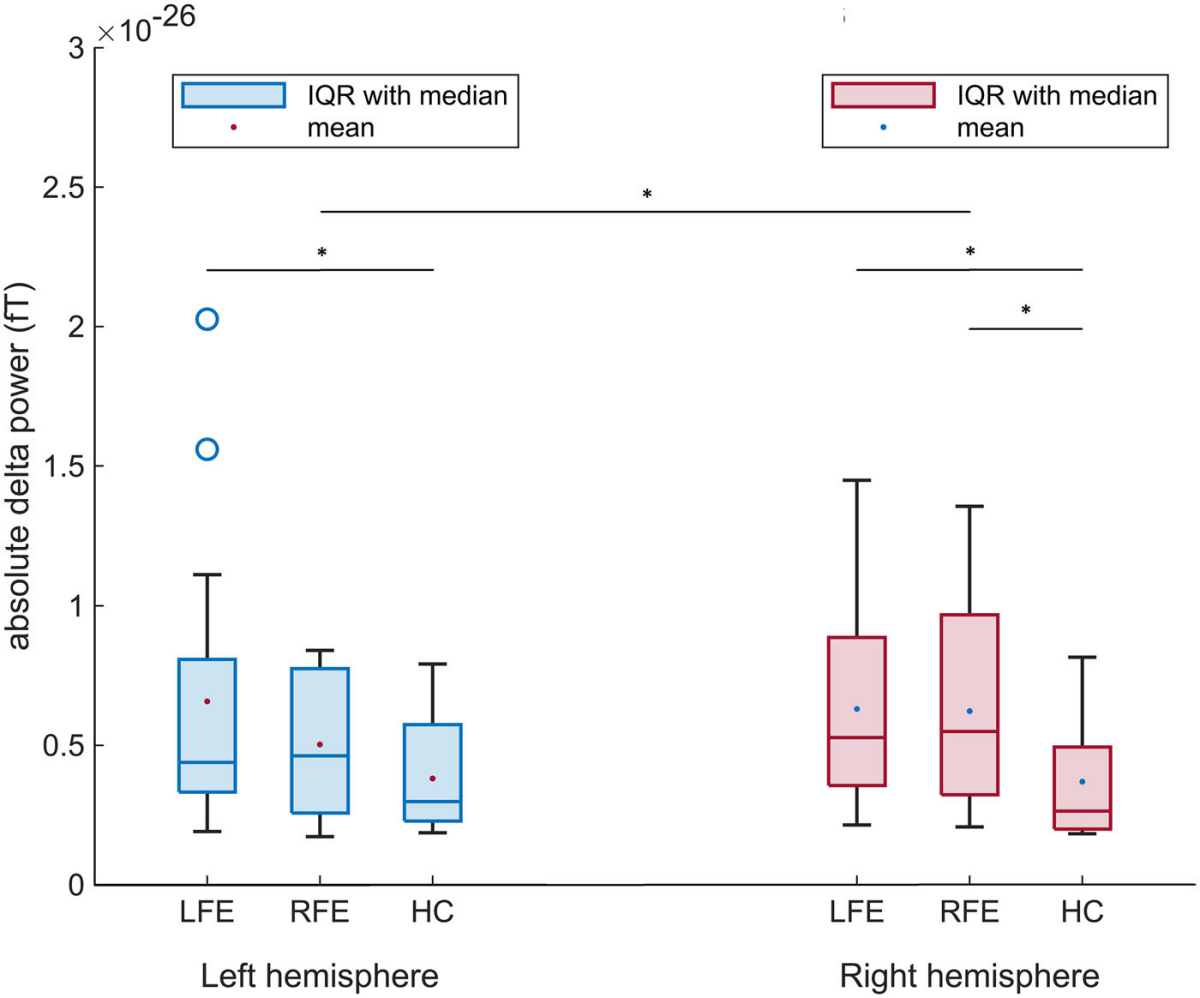
Delta power comparison between patients with left focal epilepsy (LFE), right focal epilepsy (RFE), and healthy controls (HC) as well as between the hemispheres within patients with LFE and RFE; *p < .05. fT, femtotesla; IQR, interquartile range.

Delta power was significantly higher in FE patients than in healthy controls (*U* = 75, *Z* = −1.943, *p* = .027). This was observed in both hemispheres, ipsilateral (*U* = 75, *Z* = −1.943, *p* = .027), and contralateral (*U* = 79, *Z* = −1.801, *p* = .037) to the epileptogenic zone (Figure 1). The effect size indicated a moderate effect, which was slightly higher for the ipsilateral (*r* = .324) than the contralateral (*r* = .300) hemisphere. These results were partly detected in the subgroups: Patients with LFE showed significantly higher delta power in the ipsilateral left (*U* = 44, *Z* = −1.720, *p* = .045) and the contralateral right hemisphere (*U* = 38, *Z* = −2.052, *p* = .021) compared to healthy controls. Effect sizes indicated a moderate effect, with a slightly lower effect size value for the ipsilateral left (*r* = .344) than for the contralateral right hemisphere (*r* = .410). Patients with RFE compared to healthy controls, however, showed significantly higher delta power in the ipsilateral right hemisphere (*U* = 30, *Z* = −1.760, *p* = .042), but not significantly higher delta power in the contralateral left hemisphere (*U* = 42, *Z* = −0.915, *p* > .05); with a moderate effect size in the ipsilateral right hemisphere (*r* = .384) and a small effect size in the contralateral left hemisphere (*r* = .200).

#### 3.2.2 Between patient group comparisons, left and right hemisphere

There were no significant differences in delta power between patients with LFE and RFE in neither the left (*U* = 71, *Z* = −0.597, *p* > .05) nor the right hemisphere (*U* = 79, *Z* = −0.182, *p* > .05; Figure 1). Effect sizes, however, indicated a small effect in the left hemisphere (*r* = .117), but no effect in the right hemisphere (*r* = .036).

#### 3.2.3 Lateralization of delta power

On group level, there was no clear lateralization in patients with LFE. There was neither a significant difference in delta power between the hemispheres nor an effect. In accordance with this, delta power was left lateralized (i.e., higher in the left hemisphere) in 5/15 patients (33.3%), right lateralized (i.e., higher in the right hemisphere) in 6/15 patients (40%), and not lateralized in 4/15 patients (26.7%; Table 3) on the individual level. In the group of RFE, there was a significant delta power lateralization toward the ipsilateral right hemisphere on group level (*Z* = −0.341, *p* = .016; Figure 1). The effect size indicated a large effect (*r* = .643). On individual level, RFE patients accordingly showed either right lateralization of delta power (7/11 patients, 63.6%) or no lateralization (4/11 patients, 36.4%). There was no left lateralization in this group (Table 3).

**TABLE 3:** Demographic and lateralization specific data of individual patients with left (LFE) and right focal epilepsy (RFE).

Pat. no.	Gender	Age (years)	Epilepsy duration (years)	Side of EZ	Handed‐ness	Language lateralization	Delta band power left (×10^−27^)	Delta band power right (×10^−27^)	LI delta band power	Lateralization delta band power
1	m	44	5	Left	Right	Left	3.31	3.48	−.02	Not lateralized
2	m	32	2	Left	Right	Left	8.20	9.40	−.07	Right
3	f	17	3	Left	n.a.	n.a.	15.60	12.64	.10	Left
4	m	39	19	Left	Right	Left	4.86	5.30	−.04	Not lateralized
5	f	27	12	Left	Right	Left	3.36	5.30	−.22	Right
6	m	27	1	Left	Right	n.a.	3.20	4.13	−.13	Right
7	m	53	29	Left	Right	Right	4.38	4.26	.01	Not lateralized
8	f	47	3	Left	n.a.	Left	2.83	5.91	−.35	Right
9	m	24	12	Left	Right	Left	11.12	14.49	−.13	Right
10	f	38	7	Left	Right	Left	4.54	3.81	.09	Left
11	f	26	3	Left	Right	Left	3.85	2.81	.16	Left
12	f	25	8	Left	n.a.	Left	3.47	2.66	.13	Left
13	m	26	16	Left	Right	Left	1.91	2.14	−.06	Right
14	m	19	1	Left	n.a.	n.a.	7.71	7.33	.03	Not lateralized
15	m	52	41	Left	Right	Left	20.26	10.92	.30	Left
16	f	31	5	Right	Right	Not lateralized	2.43	3.27	−.15	Right
17	m	58	39	Right	Right	Left	8.41	11.28	−.15	Right
18	m	23	2	Right	Right	Left	7.77	13.55	−.27	Right
19	m	23	7	Right	Right	Left	7.90	10.51	−.14	Right
20	m	19	1	Right	Right	Left	7.69	7.20	.03	Not lateralized
21	f	41	3	Right	Right	Left	4.62	5.49	−.09	Right
22	f	51	20	Right	Right	Left	1.73	2.15	−.11	Right
23	m	46	11	Right	Right	n.a.	6.39	5.79	.05	Not lateralized
24	f	30	9	Right	Right	Left	2.99	3.21	−.03	Not lateralized
25	f	37	2	Right	Right	Left	3.32	3.97	−.09	Right
26	m	52	51	Right	Right	Right	2.04	2.07	−.01	Not lateralized

Abbreviations: EZ, epileptogenic zone; f, female; LI, lateralization index; m, male; n.a., not available (in the case of language lateralization not available because of an insufficient acoustic window or non‐execution of investigation or in case of handedness because of non‐execution of assessment); Pat. no., patient number.

In both patient groups, there was a positive correlation of delta power between the hemispheres. In other words, that the higher the delta power in the hemisphere ipsilateral to the epileptogenic zone, the higher the delta power in the hemisphere contralateral to the epileptogenic zone (LFE: *r_s_* = .75, *p* = .001, RFE: *r_s_* = .96, *p* < .001).

#### 3.2.4 Relation between delta power and verbal fluency

There was a strong and significant negative correlation between global delta power and letter verbal fluency (*r_s_* = –.592, *p* = .008). When examining this with respect to language dominance of hemispheres, there was a strong and significant negative correlation between delta power and letter verbal fluency in the language nondominant hemisphere (*r_s_* = –.549, *p* = .028). In the language dominant hemisphere, however, a medium, but nonsignificant correlation between delta power and letter verbal fluency was found (*r_s_* = –.417). There was a small and nonsignificant correlation between global delta power and semantic verbal fluency (*r_s_* = .109).

## 4 DISCUSSION

The aim of the present study was to determine the diagnostical contribution of delta band power in unilateral FE by investigating changes in delta power and its association with cognitive functioning. Using MEG data, we compared absolute delta power, that is, the amount of MEG activity in the delta band independent of the activity in other frequency bands, in patients with unilateral FE (LFE and RFE) to healthy controls. Furthermore, we investigated the lateralization of delta power and examined the relation between delta power and the neuropsychological function verbal fluency, as verbal fluency is a cognitive function comprising either aspects of language and verbal memory in the case of semantic verbal fluency or more executive functions in the case of letter verbal fluency and thus being a representative measure of cognitive functioning.

First, following our hypothesis we found a global increase of delta power in FE patients (LFE and RFE combined) compared to healthy controls. Both patient groups differed from healthy controls: LFE patients bilaterally showed higher delta power compared to healthy controls with a more diffuse delta power dominance pattern on single patient level, whereas RFE patients revealed primarily unilateral (ipsilateral) increases in delta power. Second, there was a tendency toward delta power differences in the left hemisphere between patients with LFE and RFE, even though the delta power difference was not significant. These differences in spatial extension of delta power in FE patients might suggest functional differences between the groups. For clinical context this finding might imply that it could be relevant to distinguish not only epilepsy type but also epilepsy side when investigating medication effects, extent of neurocognitive deficits, therapy outcome etcetera. However, despite these differences, there were also similarities. Delta power co‐fluctuated between the hemispheres in both patient groups, meaning that higher delta power in one hemisphere indicated higher delta power in the contralateral hemisphere. Last, we found a negative correlation between delta power and letter verbal fluency. This suggests a negative relation between delta power and cognitive functioning in the sense that higher delta power could indicate lower cognitive information processing abilities, presumably those underlying executive functions.

### 4.1 Delta power in patients with focal epilepsy

As hypothesized, an increased global delta power was found in FE patients compared to healthy controls. This is a common finding in epilepsy patients often indicating pathological processes (Di Gennaro et al., 2003; Englot et al., 2016; Ishibashi et al., 2002; Knyazev, 2012; Laufs et al., 2006; Lin et al., 2018; Pellegrino et al., 2017). Even though there are studies showing that delta power increases occur regionally and somewhat limited to the affected hemisphere (ipsilateral to epileptogenic zone; Pellegrino et al., 2017), other recent literature indicates that delta activity can also occur bilaterally and further away from the epileptogenic zone (Lundstrom et al., 2019; Sachdev et al., 2015). In the present study, delta power increases were found not only in the affected hemisphere but also in the hemisphere contralateral to the epileptogenic zone. Delta power increases therefore presented as a bilateral phenomenon and thus not necessarily indicated specific information about the localization of the epileptogenic zone. This is underlined by the finding that for each patient—independent of the patient group—delta power was significantly correlated between both hemispheres. This could be due to the fact that this study was based on sensor‐, not source‐level data, as sensor level data is less spatially resolved, and therefore, reveals more widespread effects. However, it may also indicate ongoing network dynamic based on the high amount of cortical inter‐ and recurrent connections. The overlap of epileptic networks and intrinsic functional connectivity networks might further contribute to the global distribution of delta effects with the latter controlling basic and higher cognitive functions as suggested previously (Zhang et al., 2010). Focal and minute changes to the network caused by epileptic activity may therefore influence global brain dynamics (e.g., Carboni et al., 2020; Cataldi et al., 2013). This could explain cognitive dysfunctions related to regions beyond the epileptogenic zone that are commonly observed in patients with epilepsy (Holmes, 2015). Epileptic networks indeed have been described to have higher network efficiency (Carboni et al., 2020), a higher level of integration within the epileptic brain (Carboni et al., 2020; Samotaeva et al., 2020), higher levels of communication (Cataldi et al., 2013; Klugah‐Brown et al., 2019), and abnormal functional connectivity (de Campos et al., 2016; Cao et al., 2014; Cataldi et al., 2013; Hlinka et al., 2010). Thus increased global delta power may indicate a global information processing deficit that might cause inaccuracies in establishing precise timing which is in turn required for successful information processing (Buzsáki & Draguhn, 2004; Fries, 2015). However, delta power changes might also be a source, a correlate, or a consequence of these changes. Taken together, increased delta power in FE patients seems to be an indicator for brain‐wide abnormal communication in epileptic brains.

### 4.2 Lateralization of delta power

When investigating the lateralization of delta power, we found that patients with LFE and RFE differ in their lateralization. Whereas patients with LFE showed a similar level of delta band power in both hemispheres, patients with RFE mainly showed higher delta power in the hemisphere ipsilateral to the epileptogenic zone. In other words, patients with LFE showed a tendency to higher co‐fluctuations between delta power ipsi‐ and contralateral to the epileptogenic zone. Differences between patients with LFE and RFE are known from MEG‐ (Hsiao et al., 2015) and functional connectivity (fc)MRI‐studies (de Campos et al., 2016; Haneef et al., 2012; Samotaeva et al., 2020; Waites et al., 2006; Zhang et al., 2010). Patients with LFE and TLE generally involve wider intrinsic functional connectivity networks (de Campos et al., 2016; Haneef et al., 2012) and show a wider propagation of epileptic activity through the network as well as different brain‐wide communication patterns with more complex, bilateral changes, and a higher involvement of frontal regions (de Campos et al., 2016; Samotaeva et al., 2020; Waites et al., 2006). Physiologically, this might be due to structural differences, for example, in fiber traces and white matter (Coan et al., 2009) and suggests functional information processing differences between LFE and RFE. However, within the scope of this study, effects of epilepsy type cannot be rule out, as most of the LFE cases were TLE, whereas most of the RFE cases were FLE. Compared to FLE, TLE follows slightly different physiological principles, involves different networks and processes (Lin et al., 2020; Samotaeva et al., 2020), and shows more bilateral abnormalities (de Campos et al., 2015). However, if delta power in LFE is more of a brain‐wide phenomenon than in RFE, this may imply differences in the diagnostic value of delta power in LFE and RFE in the sense that delta power may be less reliably indicative of the epileptic hemisphere in patients with LFE than in patients with RFE.

### 4.3 Correlation between delta power and verbal fluency

We found negative correlations between delta power and lexical verbal fluency in the language dominant and nondominant hemisphere. However, there was only a small and nonsignificant association between delta power and sematic verbal fluency. Neuropsychologically, lexical verbal fluency is primarily seen as executive and frontally assigned function and thought to depend on cognitive flexibility, divergent thinking, and top–down control (Beaty et al., 2015; Conradi et al., 2020). In contrast, semantic verbal fluency has been related to frontotemporal regions and associated with verbal memory recall (Henry & Crawford, 2004; Metternich et al., 2014). Our findings, therefore, indicate that delta band increases might go along with disruptions in normal dynamics especially in networks that are involved in executive functions, but not in semantic memory retrieval. This is in accordance with various studies observing increased delta power in a number of mental disorders that are associated with executive dysfunction and rather less with verbal memory recall (Fernández et al., 2002; Newson & Thiagarajan, 2018). The stronger correlation between delta power and letter verbal fluency in the nondominant hemisphere supports this hypothesis, as executive functions are thought to rely on a distributed network involving bilateral regions (Collette et al., 2006).

Furthermore, intrinsic functional connectivity networks usually show correlated activity patterns at rest and during cognitive tasks (Sporns, 2014). Consequently, abnormalities in network's neuronal synchronization at rest may imply abnormalities during cognitive activity. Therefore, based on our findings we presume that delta power changes might be related to executive dysfunctions that are often observed in epilepsy patients in addition to the cognitive deficits explained by the epileptic focus. Those dynamics and the causality, however, are still poorly understood to date (Stretton & Thompson, 2012). As a close correlation between impairment in executive functions and the severity of network damage has been reported in the literature (Keller, Baker, Downes & Roberts, 2009), delta power in the awake resting state may be indicative of the extent of network damage.

### 4.4 Clinical application

As we found significantly higher delta power in patients with FE compared to healthy controls, delta power seems to be a marker for abnormal, pathological activity, and abnormal, disturbed brain processes in FE patients. However, the changes in delta power were not equally distributed in the patient groups with higher bilateral delta power in patients with LFE and higher unilateral delta power in patients with RFE, suggesting that delta power may be less reliably indicative of the epileptic hemisphere in patients with LFE than in patients with RFE and—as already mentioned before—that the findings might imply that it could be relevant to distinguish not only epilepsy type, but also epilepsy side when assessing medication effects, extent of neurocognitive deficits, therapy outcome etcetera. Delta power changes might thus not only be a sign of structural lesions but may also be an indicator of functional and structural network‐related changes to both hemispheres. These finding should, however, be further investigated in larger and more homogenous samples in the future. The strong negative association between delta power and letter verbal fluency supports a link between low frequency oscillations and higher order cognitive functions such as executive functions and potentially explains deficits in cognitive functions often observed beyond those explained by the epileptogenic zone.

### 4.5 Limitations and strengths

Since epilepsy is a complex and heterogeneous medical condition, and as we worked with clinical data samples, it was not possible to select homogenous patient groups. This led to slightly unbalanced groups (TLE and FLE) and resulted in small sample sizes. However, despite the small sample sizes, there were significant findings that provide a basis for future studies with more homogeneous and larger samples. Furthermore, as it is still not well understood how medication combinations affect brain dynamics, and as anti‐seizure medication (ASM) is highly individualized in patients with epilepsy, it was not possible to control for ASM effects. One way to exclude this confounder would be to investigate patients on‐ and off‐medication in the future. Last, as already mentioned, data was analyzed on sensor‐ and not on source‐level. This may limit the validity of spatial conclusions. However, as sensor data is easily accessible in clinical context and thus primarily used in clinical diagnosis, it is particularly interesting to see how delta power behaves on sensor level.

## 5 CONCLUSION

In conclusion, the study's findings, which should be further investigated in larger and more homogenous samples, confirm and provide further evidence that patients with FE have increased delta power. This is further indicative of abnormal functional and/or structural brain physiology in FE. In RFE, delta power increase was ipsilaterally accentuated, which was not reliably the case in LFE. This is diagnostically useful and indicates that delta power is not specifically bound to the diseased hemisphere, but that the lateralization of delta band power differs in respect to whether the epileptic focus is left or right. Besides, delta power correlated with functional deficits in the neuropsychological function letter verbal fluency and thus could be related to disturbances in functional networks involved in executive functions.

## AUTHOR CONTRIBUTIONS

A.M. Süß, M. Behrens, N. Conradi, R. Kienitz, S. Rampp, N. Merkel report no conflicts of interest. F. Rosenow reports personal fees and non‐financial support from UCB Pharma, personal fees from Angelini Pharma, Eisai GMBH, Jazz Pharma, LMU Munich, Medilearn India, Roche Pharma, and research grants from the German Research Foundation (DFG), BMBF—ERAPerMed Programme, European Union (FP7), Hessisches Ministerium für Wissenschaft und Kunst (LOEWE‐Programme), Hessisches Ministerium für Soziales und Integration, and the Detlev‐Wrobel‐Fonds for Epilepsy Research Frankfurt, and grants by the Chaja Foundation, Dr. Schär Deutschland GmbH, Vitaflo Deutschland GmbH, Nutricia Milupa GmbH, and Desitin Arzneimittel GmbH, Hamburg, outside the submitted work.

### PEER REVIEW

The peer review history for this article is available at https://publons.com/publon/10.1002/brb3.3257

## Data Availability

The data that support the findings of this study are available on request from the corresponding authors, A.M. Süß or N. Merkel. The data is not publicly available due to their containing information that could compromise the privacy of research participants.
